# Fractional exhaled nitric oxide in idiopathic pulmonary arterial hypertension and mixed connective tissue disease complicating pulmonary hypertension

**DOI:** 10.1186/s12890-024-03004-x

**Published:** 2024-04-23

**Authors:** Jianhua Xu, Xingxing Sun, Yuan Cao, Hanqing Zhu, Wenlan Yang, Jinming Liu, Jian Guo

**Affiliations:** 1 Department of Pulmonary Function Test, Shanghai Pulmonary Hospital, School of Medicine, Tongji University, 200092 Shanghai, China; 2 Department of Pulmonary Circulation, Shanghai Pulmonary Hospital, School of Medicine, Tongji University, 200092 Shanghai, China

**Keywords:** FeNO, IPAH, MCTD-PH, Correlation

## Abstract

**Background:**

Fractional exhaled nitric oxide (FeNO) has been extensively studied in various causes of pulmonary hypertension (PH), but its utility as a noninvasive marker remains highly debated. The objective of our study was to assess FeNO levels in patients with idiopathic pulmonary arterial hypertension (IPAH) and mixed connective tissue disease complicating pulmonary hypertension (MCTD-PH), and to correlate them with respiratory functional data, disease severity, and cardiopulmonary function.

**Methods:**

We collected data from 54 patients diagnosed with IPAH and 78 patients diagnosed with MCTD-PH at the Shanghai Pulmonary Hospital Affiliated to Tongji University. Our data collection included measurements of brain natriuretic peptide (pro-BNP), cardiopulmonary exercise test (CPET), pulmonary function test (PFT), impulse oscillometry (IOS), and FeNO levels. Additionally, we assessed World Health Organization functional class (WHO-FC) of each patient.

**Results:**

(1) The fractional exhaled concentration of nitric oxide was notably higher in patients with IPAH compared to those with MCTD-PH. Furthermore, within the IPAH group, FeNO levels were found to be lower in cases of severe IPAH compared to mild IPAH (*P* = 0.024); (2) In severe pulmonary hypertension as per the WHO-FC classification, FeNO levels in IPAH exhibited negative correlations with FEV1/FVC (Forced Expiratory Velocity at one second /Forced Vital Capacity), MEF50% (Maximum Expiratory Flow at 50%), MEF25%, and MMEF75/25% (Maximum Mid-expiratory Flow between 75% and 25%), while in severe MCTD-PH, FeNO levels were negatively correlated with R20% (Resistance at 20 Hz); (3) ROC (Receiving operator characteristic curve) analysis indicated that the optimal cutoff value of FeNO for diagnosing severe IPAH was 23ppb; (4) While FeNO levels tend to be negatively correlated with peakPETO2(peak end-tidal partial pressure for oxygen) in severe IPAH, in mild IPAH they had a positive correlation to peakO2/Heart rate (HR). An interesting find was observed in cases of severe MCTD-PH, where FeNO levels were negatively correlated with HR and respiratory exchange ratio (RER), while positively correlated with O2/HR throughout the cardiopulmonary exercise test.

**Conclusion:**

FeNO levels serve as a non-invasive measure of IPAH severity. Although FeNO levels may not assess the severity of MCTD-PH, their significant makes them a valuable tool when assessing severe MCTD-PH.

## Introduction

PH is a rare and progressive disease that can have several potential etiologies and is associated with respiratory and cardiovascular diseases. It is characterized by elevated lung vascular resistance, ultimately leading to premature death [1]. The assessment of treatment efficacy in PAH relies on prognostic parameters such as pro-BN, WHO-FC, 6-minute walk test (6MWT), echocardiographic findings, among others. However, these tools are insufficient for making decisions regarding prognosis and severe PH. Therefore, there is still a need for noninvasive prognostic biomarkers in PH patients [2].

Previous studies have demonstrated that IPAH and MCTD-PH share similar pathological features, including medial hypertrophy of muscular and elastic arteries, dilation and intimal atheroma of elastic pulmonary arteries, as well as right ventricular hypertrophy [3]. After IPAH, PAH-CTD is the second most prevalent type of PAH in western countries [4]. Peripheral airway obstruction occurs both in IPAH and CTD-PAH patients, while lower DLCO (the diffusion of lung carbon monoxide) levels are found in CTD-PAH patients alone [5, 6]. In SSc-PAH (systemic sclerosis-PH) patients, there is a frequent reduction in DLCO with normal FVC. The reduction of DLCO during the follow-up of SSc patients could be considered as a sign of possible PAH presence. Given the association between peripheral airways affected by the development of PH following the pulmonary vasculature pathogenesis hypothesis suggests that it may impact pulmonary function [7]. Nitric oxide (NO) signaling deficiency plays a crucial role in the pathogenesis of PH [8]. While endothelial cells produce high levels of NO with only a small fraction being exhaled into the lungs; NO regulates the response to hypoxia through vasodilation and smooth muscle cell proliferation control [9, 10]. As an important gaseous mediator involved in PH pathogenesis synthesized from L-arginine amino acid via nitric oxide synthase enzymes which exist in three isoforms: neuronal NOS (nNOS), endothelial NOS (eNOS), inducible NOS (iNOS) [11]. It has been reported that NO exhibits concentration-dependent effects: vasodilative effect in low level or oxidation as the main pathway while in higher level, the pathophysiology of PH has been linked to the disruption of the NO pathway [12]. eNOS is mainly expressed in the vascular endothelium and it is the main source for NO in the pulmonary circulation [13]. Some studies have reported increased eNOS expression in PAH patients and in animal models of PAH [14, 15]. Several studies have described a decrease in NO concentration in exhaled breath (FeNO) in PAH, particularly the idiopathic variety (IPAH), FeNO correlated with hemodynamic severity of disease and prognosis and has been shown to increase in response to therapy [16].

FeNO’s usefulness as a noninvasive marker remains highly controversial. Therefore, the aim of this study was to determine FeNO values in patients with IPAH and MCTD-PH, and evaluate correlation between FeNO and other relevant parameters.

## Patients and methods

### Ethical approval

The study protocol underwent a thorough review and received approval from the Ethics Committee of Shanghai Pulmonary Hospital. All methods, including Echocardiography, pro-BNP, right-heart catheterization (RHC), CPET, PFT, FeNO measurements, and IOS were conducted in strict accordance with the applicable guidelines and regulations.

### Patients

This retrospective study included a total of 54 patients diagnosed with IPAH and 78 patients with MCTD-PH, who were examined in Shanghai Pulmonary Hospital between January 2018 and December 2021. The main exclusion criteria for PAH encompassed smoking with in the preceding six months, respiratory tract infection in the last six weeks, patients with exacerbations or patients with well-controlled underlying chronic disease such as COPD, bronchiectasis and asthma. and the presence of secondary causes of IPAH such as heart embolism, lupus, HIV, and liver cirrhosis; usage of L-arginine, nitrates, or phosphodiesterase inhibitors; and PH caused other diseases other than MCTD-PH.

Based on WHO-FC at enrollment, all PAH patients were categorized into two groups: mild (Class I-II) and severe (Class III-IV).

### PFT

We conducted pulmonary function tests, which included spirometry and IOS, using standard equipment (Masterscreen-PFT, Jaeger crop, Hoechberg, Germany; Masterscreen-IOS, Jaeger crop, Hoechberg, Germany). The following parameters were determined by standard procedures: FVC, FEV1, FEV1/FVC ratio, MEF25%, MEF50%, and MMEF75/25% [17]. Oscillometry measurements, which included R5, R5%, R20, R20%, and Xrs at 5 Hz (X5), were recorded prior to spirometry. The duration of the IOS test was between 30 and 45 s. Spirometry measurements were carried out following the standards recommended by The European Respiratory Society [18].

### FeNO

FeNO levels were assessed using an electrochemical breath analyzer from Shangwo Medical Electronics, Wuxi, China, operated by a specialized team. The FeNO measurements in this study adhered to the FeNO operation standards recommended by the American Thoracic Society (ATS) and European Respiratory Society (ERS). To minimize potential interference from food or beverages, patients were given instructions not to consume any food or drink for a minimum of 2 h prior to the measurement. No smoking 1 h before the test. FeNO measurements were conducted prior to pulmonary function tests. Patients were instructed to maintain steady and smooth breathing for at least two breath cycles, exhaling at a constant airflow of 50 mL/s for 10 s. FeNO levels were measured thrice for each patient under stable conditions, ensuring that the difference between measurements did not exceed 10%.

### Right heart catheterization

All patients with PAH underwent RHC. A True Size Thermodilution Catheter (Edward 774) was inserted through the left cubital vein. Hemodynamic measurements encompassed mean right atrium pressure (mRAP), mean pulmonary arterial pressure (mPAP) and pulmonary artery wedge pressure (PAWP). Cardiac output (CO) was determined using the thermodilution method. Cardiac output (CO) was determined using the thermodilution method. Pulmonary vascular resistance (PVR) was calculated employing standard formulas: PVR = (mPAP– PAWP)/CO.

### CPET

CPET was conducted using an electromagnetically braked cycle ergometer (Master Screen CPX, Jaeger crop, Hoechberg, Germany) to record gas exchange data at 10-second intervals through a breath-by-breath system. The protocol comprised four stages: a 3-minute rest phase, a 3-minute unloaded phase, an incremental phase, and a 5-minute recovery phase. Participants were allowed to discontinue the test if they experienced any discomfort such as fatigue, dyspnea or chest tightness. An appropriate model (10 W/min, 15 W/min and 20 W/min) was selected based on the patient’s clinical condition and pulmonary function test results. During the unloaded and incremental phases, participants were instructed to pedal at a rate of 55–60 revolutions per minute. Measurements including oxygen pulse (VO2 /HR), end-tidal partial pressure for carbon dioxide (PETCO2), PETO2, HR, and RER were recorded and calculated. Anaerobic threshold (AT), representing the onset of anaerobic metabolism, was determined using both the V-slope method and independent assessment by two experienced investigators with extensive clinical and scientific research experience in CPET.

### Statistical analysis

Statistical analysis was conducted using SPSS 22.0 and GraphPad Prism 5 software. Data were presented as either as mean ± SD or frequency and median (interquartile range). The normality of distributions was assessed using the Shapiro–Wilk test. Proportional differences were evaluated using the Chi-square test, while differences between two groups were analyzed using an independent sample t-test. The Wilcoxon Mann-Whitney test was employed for data that did not adhere to normal distribution. Correlation coefficients among pulmonary function parameters, hemodynamic parameters, CPET results, and serum markers were determined using Spearman correlation coefficients. To assess the diagnostic value of FeNO for severe PH in IPAH, ROC curve was utilized. A *p*-value less than 0.05 was considered statistically significant.”

## Results

### Clinical characteristics in IPAH and MCTD-PH

The demographic data and baseline lung function test results for patients with IPAH and MCTD-PH are presented in Table 1. A total of 54 IPAH and 78 MCTD-PH patients were included in the study. No statistically significant differences were observed in age and BMI (*P* > 0.05) between the IPAH and MCTD-PH groups; both diseases were more prevalent among women. Pro-BNP levels did not significantly differ between IPAH and MCTD-PH (*P* = 0.333).

FeNO levels were found to be higher in IPAH compared to MCTD-PH (*P* = 0.003). Notably, statistically significant differences in FVC and FEV1 (*P* < 0.0001) were observed between IPAH and MCTD-PH, with FVC and FEV1 being higher in the IPAH group. Furthermore, statistically significant differences were noted in R5 (*P* = 0.002), R20 (*P* = 0.001), and R20% (*P* = 0.036) between IPAH and MCTD-PH, with R5, R20, and R20% being lower in IPAH compared to MCTD-PH.

**Table 1:** Baseline Characteristics and Lung Function of IPAH and MCTD-PH

Factors	IPH (*N* = 54)	MCTD-PH (*N* = 78)	*P*
Age, years	42.00 ± 14.16	44.28 ± 14.03	*P* = 0.362
BMI, kg/m2	23.79 ± 3.47	22.62 ± 3.40	*P* = 0.056
Sex, M/F	19/35	3/75	
WHO functional class			
Class I, I-II, II	23	26	
Class II-III, III	25	49	
Class III-IV, IV	3	6	
NT-ProBNP, pg/ml	502.1 (77.35–1248)	512.3 (164.9–1678)	*P* = 0.333
FeNO, (ppb)	19 (14–27)	15 (8.75–21.5)	*P* = 0.003
PFT			
FVC, L	2.93 ± 0.79	2.33 ± 0.64	*P* < 0.0001
FEV1, L	2.37 (1.94–2.76)	2.05 (1.50–2.34)	*P* < 0.0001
FEV1/FVC	82.18 ± 5.64	83.53 ± 6.99	*P* = 0.239
MEF50%	84.05 (67.83-104.35)	76.40 (61.48–96.90)	*P* = 0.122
MEF25%	62.45 (45.28–83.08)	60.55 (41.48–78.95)	*P* = 0.485
MMEF75/25%	68.17 ± 21.33	61.62 ± 21.28	*P* = 0.084
IOS			
R5, cmH2O/(L/s)	3.79 (3.07–4.60)	4.32 (3.71–5.71)	*P* = 0.002
R5%	110.90 (93.75–127.80)	117.10 (101.75–153.20)	*P* = 0.052
R20, cmH2O/(L/s)	2.75 (2.45–3.54)	3.47 (2.63–4.39)	*P* = 0.001
R20%	95.70 (85.65-120.15)	110.80 (87.75-140.95)	*P* = 0.036

The data are presented as mean ± SD, frequency and median (interquartile range). Age and BMI were analyzed by Chi-square test, Wilcoxon Mann-Whitney test was used for data that did not conform to normality of distributions. BMI = body mass index; BNP = brain natriuretic peptide; FeNO = fractional exhaled nitric oxide; PFT = pulmonary function tests; FVC = forced vital capacity; FEV1 = forced expiratory volume in 1 s; MEF = maximal expiratory flow; MMEF = maximum midexpiratory flow; IOS = impulse oscillation; R5 = R at 5 Hz; R20 = R at 20 Hz.

### Association between fractional exhaled nitric oxide and clinical characteristics in patients with IPAH

**Fig. 1:**
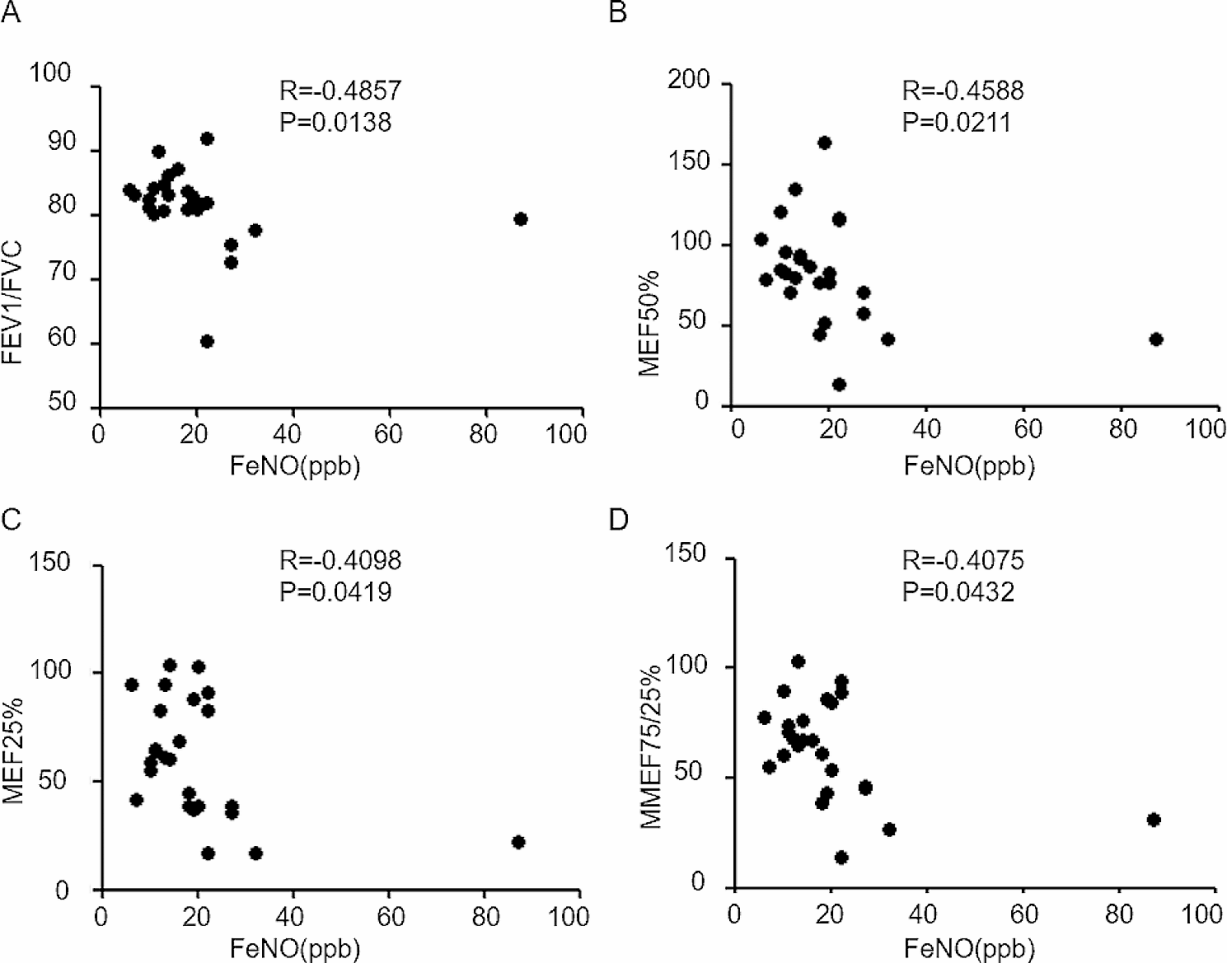
Association between FeNO levels and PTF parameters in IPAH patients with severe PH. **A**. Association between FeNO levels and FEV1/FVC. **B.** Association between FeNO levels and MEF50%. **C**. Association between FeNO levels and MEF25%. **D.** Association between FeNO levels and MMEF75/25%. See Tables 1 and 2 legend for expansion of abbreviations

The relationship between FeNO levels and clinical parameters in patients with IPAH is presented in Table 2. Notably, FeNO levels exhibited a negative correlation with FEV1/FVC in IPAH (*R*=-0.319, *P* = 0.019), particularly in cases of severe IPAH (*R*=-0.485, *P* = 0.014). Additionally, it is noteworthy that FeNO levels demonstrated negative correlations with MEF50%, MEF25%, and MMEF75/25% in severe IPAH (*R*=-0.459, *P* = 0.021; *R*=-0.410, *P* = 0.042; *R*=-0.408, *P* = 0.043) (Fig. 1). However, FeNO levels did not show significant correlations with FEV1/FVC, MEF50%, MEF25%, and MMEF75/25% in mild IPAH (*R*=-0.257, *P* = 0.206; *R*=-0.062, *P* = 0.763; *R*=-0.002, *P* = 0.991; *R*=-0.074, *P* = 0.721). Given the numerous parameters in CPET, we selected specific parameters for inclusion in the table. FeNO levels displayed a negative correlation with ATHR (anaerobic threshold heart rate) in mild IPAH (*R*=-0.420, *P* = 0.033), whereas FeNO levels were positively correlated with PeakO2/HR (peak oxygen consumption divided by peak heart rate) in mild IPAH (*R* = 0.451, *P* = 0.021). Furthermore, FeNO levels showed a negative correlation with PeakPETO2 in IPAH (*R*=-0.392, *P* = 0.005), particularly in cases of severe IPAH (*R*=-0.468, *P* = 0.024). However, FeNO levels exhibited a positive correlation with PeakPETCO2 in IPAH (*R* = 0.293, *P* = 0.039).

**Table 2:** Relationship between FeNO levels and clinical parameters in patients with IPAH.

Factors	IPAH: all (54)		IPAH: mild (26)		IPAH: severe (25)	
	R	*P*	R	*P*	R	*P*
PFT						
FVC, L	-0.101	0.469	-0.235	0.248	-0.122	0.560
FEV1, L	-0.141	0.309	-0.219	0.282	-0.169	0.419
FEV1/FVC	-0.319	0.019	-0.257	0.206	-0.485	0.014
MEF50%	-0.206	0.136	-0.062	0.763	-0.459	0.021
MEF25%	-0.191	0.167	-0.002	0.991	-0.410	0.042
MMEF75/25%	-0.172	0.214	-0.074	0.721	-0.408	0.043
IOS						
R5, cmH2O/(L/s)	-0.009	0.950	-0.092	0.663	0.012	0.956
R5%	-0.077	0.583	-0.142	0.497	0.080	0.705
R20, cmH2O/(L/s)	-0.117	0.403	-0.149	0.479	-0.163	0.436
R20%	-0.217	0.119	-0.205	0.325	-0.248	0.232
CPET						
restHR	0.136	0.345	0.020	0.924	0.071	0.742
restO2/HR	0.043	0.768	0.266	0.768	-0.065	0.764
restRER	0.073	0.616	-0.026	0.900	0.032	0.881
warmHR	-0.098	0.497	-0.250	0.219	0.026	0.906
warmO2/HR	0.091	0.531	0.317	0.114	-0.089	0.679
warmRER	0.242	0.090	0.287	0.156	0.279	0.188
ATHR	-0.135	0.349	-0.420	0.033	-0.070	0.746
ATO2/HR	0.232	0.105	0.386	0.052	-0.050	0.817
ATRER	0.098	0.499	0.042	0.840	0.397	0.055
PeakHR	-0.150	0.298	-0.266	0.189	-0.332	0.113
PeakO2/HR	0.269	0.059	0.451	0.021	-0.065	0.765
PeakRER	-0.051	0.725	-0.114	0.581	-0.160	0.454
PeakPETO2, mmHg	-0.392	0.005	-0.295	0.144	-0.468	0.024
PeakPETCO2, mmHg	0.293	0.039	0.245	0.586	0.270	0.202

Correlation coefficients were expressed as Spearman correlation coefficients. CPET=cardiopulmonary exercise test; O2/HR= oxygen consumption divided by peak heart rate; HR=Heart rate; RER=Respiratory exchange ratio; PETO2=End-tidal partial pressure for oxygen; PETCO2=end-tidal partial pressure for carbon dioxide. See Table 1 legend for expansion of abbreviations.

**Fig. 2:**
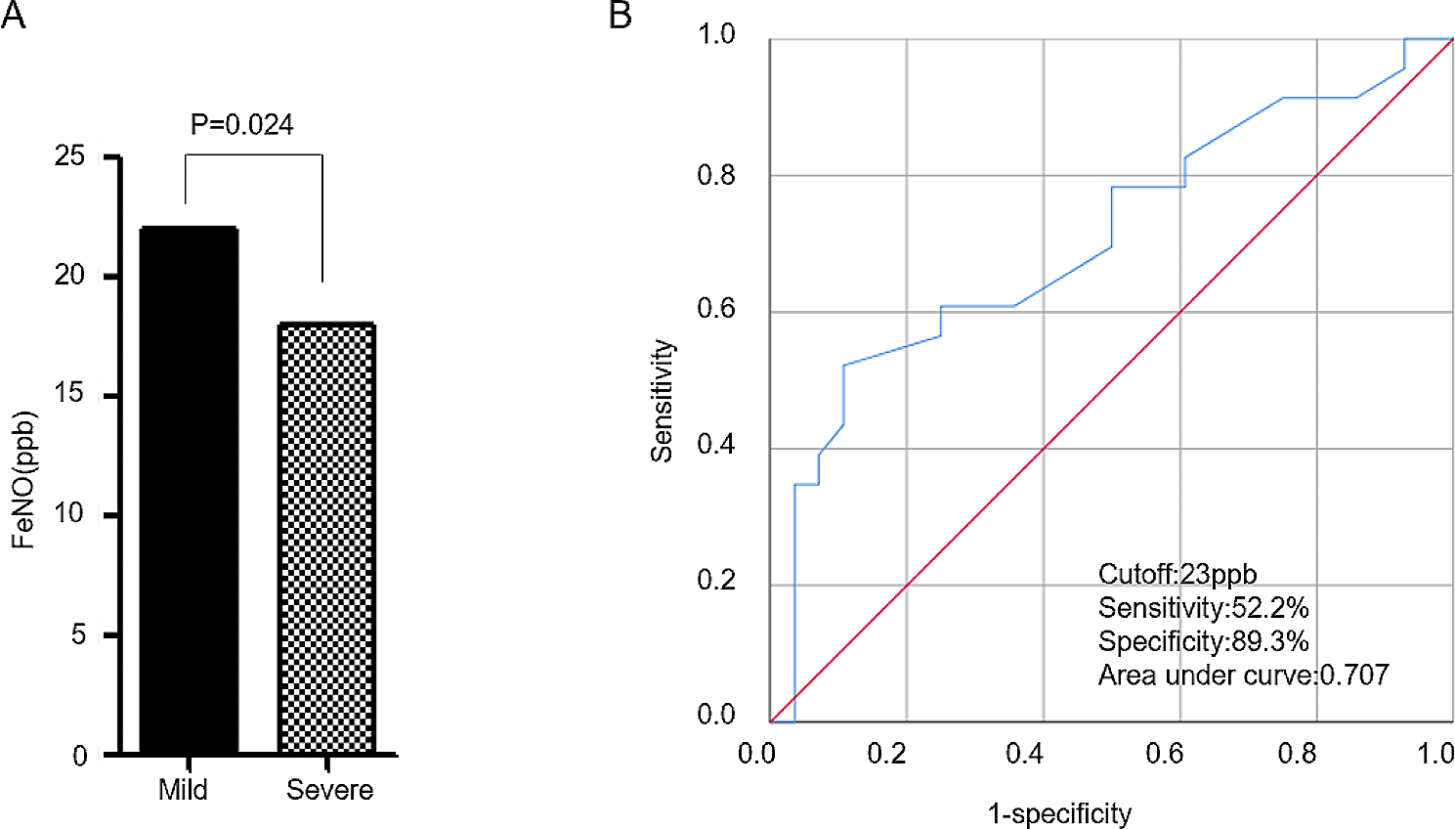
The diagnostic value of FeNO levels in IPAH. **(A)** FeNO levels in patients with mild and severe PH in IPAH (Graphing replicates or error bars plot: Median). **(B)** ROC curve showing the diagnostic value of FeNO level for severe PH in IPAH patients. See Table 1 legend for expansion of abbreviations

To assess the diagnostic value of FeNO levels in identifying severe PH in patients with IPAH, we employed ROC curve analysis. Initially, we compared FeNO levels between patients with mild and severe PH in the IPAH group. Notably, FeNO levels were found to be lower in patients with severe PH (*P* = 0.024) (Fig. 2A). Subsequently, ROC curve analysis yielded an area under the curve (AUC) of 0.707 (95% confidence interval (CI) = 0.560–0.855). The optimal cutoff value for FeNO in diagnosing IPAH with severe PH was determined to be 23 ppb, with a sensitivity of 52.2% and specificity of 89.3% (Fig. 2B). These results indicate that the potential diagnostic utility of FeNO levels in IPAH patients with severe PH.

### Association between fractional exhaled nitric oxide and clinical characteristics in patients with MCTD-PH

**Fig. 3:**
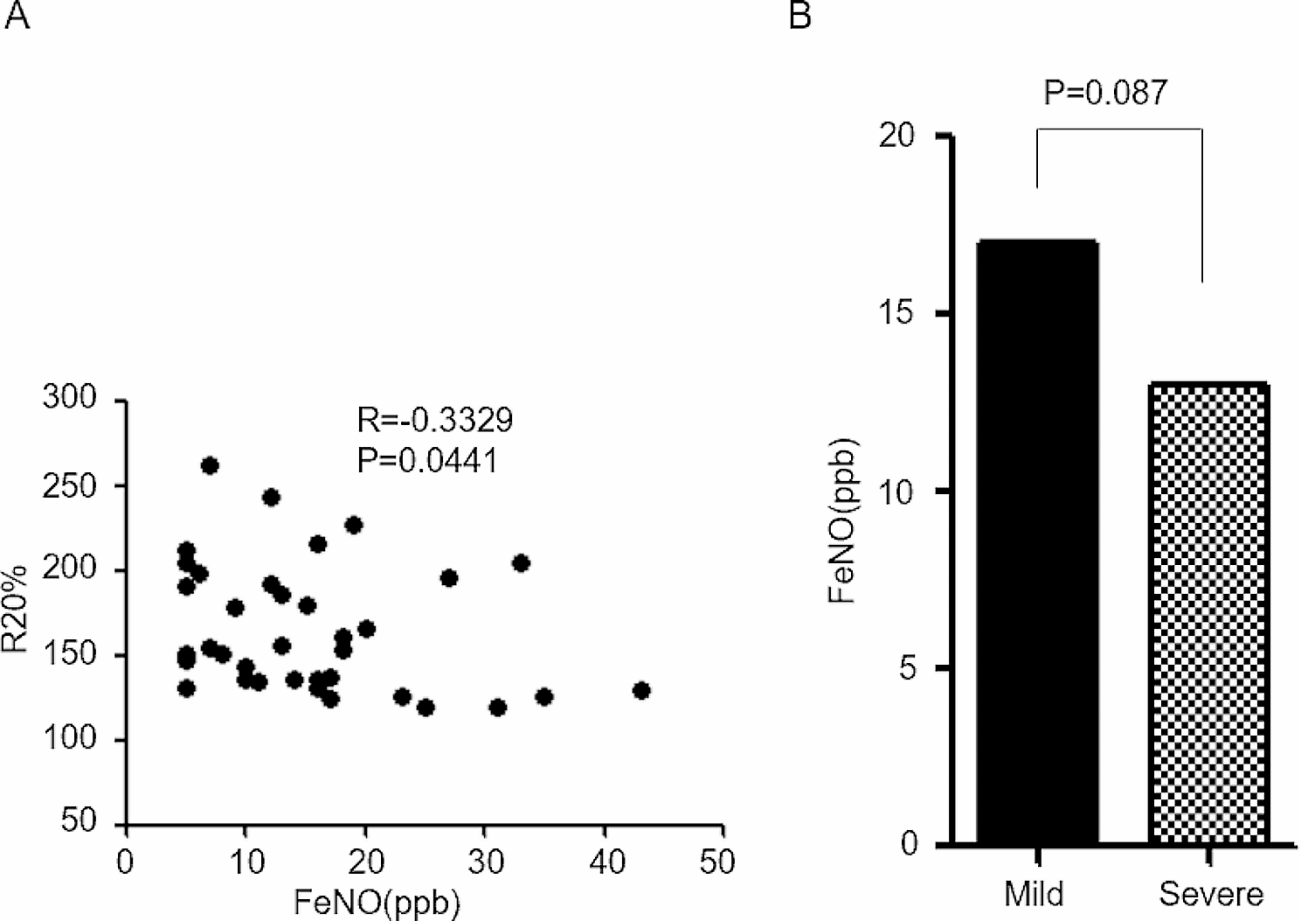
**(A)** Association between FeNO levels and R20% in severe PH patients with MCTD. **(B)** FeNO levels in patients with mild and severe PH in MCTD-PH (Graphing replicates or error bars plot: Median). See Table 1 legend for expansion of abbreviations

The relationship between FeNO levels and clinical parameters in patients with MCTD-PH is presented in Table 3. An intriguing observation is that FeNO levels exhibited a negative correlation solely with R20% (*R*=-0.240, *P* = 0.036), particularly in cases of severe MCTD-PH (*R*=-0.333, *P* = 0.044, as depicted in Fig. 3A). FeNO levels did not display significant correlations with other lung function parameters in this context. Furthermore, during the four periods of CPET, the correlation between FeNO levels and parameters remained consistent in severe MCTD-PH. Specifically, FeNO levels were negatively correlated with resting heart rate (restHR), warming-up heart rate (warmHR), anaerobic threshold heart rate (ATHR), and peak heart rate (PeakHR) in severe MCTD-PH (*R*=-0.390, *P* = 0.016; *R*=-0.465, *P* = 0.003; *R*=-0.516, *P* = 0.001; *R*=-0.485, *P* = 0.002). FeNO levels also exhibited negative correlations with resting respiratory exchange ratio (restRER), warming-up RER (warmRER), anaerobic threshold RER (ATRER), and peak RER (PeakRER) in severe MCTD-PH (*R*=-0.416, *P* = 0.009; *R*=-0.459, *P* = 0.004; *R*=-0.467, *P* = 0.004; *R*=-0.361, *P* = 0.026). However, FeNO levels displayed positive correlations with resting oxygen consumption divided by heart rate (restO2/HR), warming-up O2/HR (warmO2/HR), anaerobic threshold O2/HR (ATO2/HR), and peak O2/HR in severe MCTD-PH (*R* = 0.527, *P* = 0.001; *R* = 0.567, *P* = 0.000; *R* = 0.477, *P* = 0.003; *R* = 0.456, *P* = 0.004). There was no statistically significant difference in FeNO levels between patients with mild and severe PH in the MCTD group (*P* = 0.087, Fig. 3B), hence we did not perform ROC curve analysis.

**Table 3:** Relationship between FeNO levels and clinical parameters in patients with MCTD-PH

Factors	MCTD-PH: all (78)		MCTD-PH: mild (33)		MCTD-PH: severe (38)	
	R	*P*	R	*P*	R	*P*
PFT						
FVC, L	0.081	0.484	0.086	0.636	-0.024	0.886
FEV1, L	0.042	0.717	0.000	1.000	-0.045	0.789
FEV1/FVC	-0.166	0.147	-0.059	0.746	-0.224	0.176
MEF50%	-0.133	0.245	-0.045	0.804	-0.227	0.170
MEF25%	-0.109	0.343	-0.068	0.705	-0.146	0.383
MMEF75/25%	-0.103	0.369	-0.017	0.924	-0.243	0.142
IOS						
R5, cmH2O/(L/s)	-0.033	0.208	0.060	0.741	-0.012	0.946
R5%	-0.056	0.773	0.042	0.817	-0.015	0.932
R20, cmH2O/(L/s)	-0.215	0.060	-0.007	0.969	-0.292	0.080
R20%	-0.240	0.036	0.020	0.910	-0.333	0.044
CPET						
restHR	-0.318	0.005	-0.219	0.222	-0.390	0.016
restO2/HR	0.279	0.014	-0.015	0.935	0.527	0.001
restRER	-0.215	0.058	-0.118	0.513	-0.416	0.009
warmHR	-0.383	0.001	-0.234	0.190	-0.465	0.003
warmO2/HR	0.380	0.001	-0.030	0.866	0.567	0.000
warmRER	-0.165	0.149	-0.008	0.965	-0.459	0.004
ATHR	-0.416	0.000	-0.269	0.137	-0.516	0.001
ATO2/HR	0.339	0.003	0.038	0.837	0.477	0.003
ATRER	-0.252	0.030	-0.070	0.705	-0.467	0.004
PeakHR	-0.290	0.010	-0.167	0.354	-0.485	0.002
PeakO2/HR	0.355	0.001	0.079	0.661	0.456	0.004
PeakRER	-0.120	0.295	-0.009	0.959	-0.361	0.026
PeakPETO2, mmHg	-0.133	0.245	-0.028	0.878	-0.114	0.497
PeakPETCO2, mmHg	0.150	0.191	0.176	0.326	-0.076	0.649

Correlation coefficients were expressed as Spearman correlation coefficients. See Tables 1 and 2 legend for expansion of abbreviations.

## Discussion

In this study, we measured FeNO levels in patients with IPAH and MCTD-PH. Upon examining the demographic data and baseline lung function test results of both patient groups, we found no statistically significant differences in age and BMI (*P* > 0.05). Additionally, both diseases exhibited a higher prevalence among women, and there was no statistically significant difference in NT-ProBNP levels (*P* > 0.05). However, notable differences emerged in FeNO levels (*P* = 0.003) and lung function parameters (*P* < 0.05) when comparing IPAH to MCTD-PH. Specifically, FeNO levels, FVC, and FEV1 were all significantly higher in IPAH patients compared to those with MCTD-PH (*P* < 0.05). Conversely, parameters such as R5, R20, and R20% were significantly lower in IPAH patients compared to MCTD-PH patients (*P* < 0.05). These findings suggest that airway restriction and obstruction were more pronounced in MCTD-PH patients compared to those with IPAH.

MEF50%, MEF25%, and MMEF75/25% have been widely employed by various authors as sensitive indicators of peripheral airflow limitation in conditions such as chronic obstructive pulmonary disease, occupational lung disease, and primary pulmonary hypertension [19–21]. Previous studies have also suggested the occurrence of peripheral airway obstruction in IPAH [7, 22], Our study results align with these previous findings, as we observed that FeNO levels were significantly negatively correlated with FVC/FEV1, MEF50%, MEF25%, and MMEF75/25% in severe IPAH (*R*=-0.459, *P* = 0.021; *R*=-0.410, *P* = 0.042; *R*=-0.408, *P* = 0.043). Moreover, FeNO levels displayed a negative correlation with PeakPETO2 in severe IPAH (*R*=-0.468, *P* = 0.024). Interestingly, FeNO levels exhibited a positive correlation with PeakPETCO2 in IPAH (*R* = 0.293, *P* = 0.039). Subsequently, ROC curve analysis revealed that the optimal cutoff value for FeNO in diagnosing IPAH with PH was determined to be 23 ppb. Additionally, FeNO levels were lower in severe PH patients compared to mild PH patients (*P* = 0.024). The role of endogenous NO production in PH remains uncertain and highly debated. Some studies have reported lower FeNO levels in PAH patients compared to healthy individuals [23, 24], while others have found FeNO levels to be similar between healthy subjects, IPAH patients, and patients with PAH associated with scleroderma [25, 26]. Notably, a study specifically investigated FeNO as a biomarker for IPAH or treatment efficacy and concluded that FeNO is not a reliable biomarker for either [27]. They only focused on the differences in FeNO levels at baseline between IPAH patients and control subjects, as well as changes in FeNO levels during three months of treatment, finding that FeNO levels did not correlate with other disease measures. In our study, we delved further into the relationship between FeNO and lung function, as well as cardiopulmonary function in severe IPAH. The results of our investigation suggest that FeNO levels can indeed aid in the diagnosis of IPAH patients with severe PH, with higher FeNO levels indicating a greater degree of peripheral airway obstruction in severe IPAH.

IOS is gaining prominence in pulmonary function examinations as it assesses respiratory impedance across a spectrum of frequencies ranging from 5 to 35 Hz. Within this assessment, R5 represents the overall airway resistance, while R20 specifically denotes the resistance of the larger airways [28, 29]. It’s worth noting that previous studies have indicated no significant correlation between FeNO and abnormal PFTs [30, 31]. It is intriguing to note that in our study, FeNO levels exhibited a unique pattern of correlation. Specifically, FeNO levels displayed a negative correlation solely with R20% (*R*=-0.240, *P* = 0.036), particularly pronounced in severe MCTD-PH cases (*R*=-0.333, *P* = 0.044). In cases of severe MCTD-PH where there was higher resistance in the large airways (R20%), FeNO levels were lower. Notably, FeNO levels did not show significant correlations with other lung function parameters. This finding stands in contrast to the behavior of FeNO in IPAH, where FeNO levels were not significantly correlated with IOS parameters (*P* > 0.05).

In addition, FeNO levels displayed a series of correlations in severe MCTD-PH. Specifically, FeNO levels exhibited negative correlations with restHR, warmHR, ATHR, and PeakHR in severe MCTD-PH (*R*=-0.390, *P* = 0.016; *R*=-0.465, *P* = 0.003; *R*=-0.516, *P* = 0.001; *R*=-0.485, *P* = 0.002). Furthermore, FeNO levels demonstrated negative correlations with restRER, warmRER, ATRER, and PeakRER in severe MCTD-PH (*R*=-0.416, *P* = 0.009; *R*=-0.459, *P* = 0.004; *R*=-0.467, *P* = 0.004; *R*=-0.361, *P* = 0.026). Conversely, FeNO levels displayed positive correlations with resting oxygen consumption divided by restO2/HR, warmO2/HR, ATO2/HR, and PeakO2/HR in severe MCTD-PH (*R* = 0.527, *P* = 0.001; *R* = 0.567, *P* = 0.000; *R* = 0.477, *P* = 0.003; *R* = 0.456, *P* = 0.004). Additionally, no statistically significant difference in FeNO levels was observed between patients with mild and severe PH in the MCTD group (*P* = 0.087). The findings illuminate a significant pattern of correlations related to FeNO levels. FeNO levels displayed negative correlations with HR and RER, while they exhibited positive correlations with restO2/HR, both in resting and active states. This suggests a potential relationship between FeNO levels and heart function. Although FeNO levels may not serve as a direct diagnostic tool for identifying severe PH in MCTD-PH patients, our results indicate that FeNO levels are associated with R20% and cardiopulmonary function in cases of severe PH. As such, FeNO levels hold promise as a valuable marker for identifying severe MCTD-PH.

The contrasting correlations observed between FeNO and various parameters in IPAH and MCTD-PH may be attributed to several factors. A prior study demonstrated that patients with pulmonary arterial hypertension related to scleroderma (PAH-Scl) exhibited a lower mean pulmonary artery pressure compared to IPAH patients, despite both groups showing similar levels of cardiac dysfunction. Interestingly, echocardiography findings indicated comparable degrees of right ventricular dysfunction in both groups, whereas a predominance of left heart dysfunction was evident in patients with PAH-Scl [32]. Interestingly, there was no suggestion that FeNO levels were lower in individuals with higher pulmonary artery pressure or vascular resistance. In fact, our findings revealed that FeNO levels was not significantly correlated with RHC parameters in both patient groups (the data were not listed in the table, *P* > 0.05). However, in the case of severe MCTD-PH, FeNO levels did exhibit a significant correlation with HR, RER, and O2/HR. These results indicate that the association between FeNO levels and pulmonary artery pressure or hemodynamic factors warrants further investigation. Additionally, it’s worth noting that higher levels of dimethylarginine, a potent inhibitor of eNOS, have been shown to reduce NO production by eNOS in patients with IPAH [33].

NO formation has been found to be elevated in patients with primary limited SSc; however, the nitration of proteins and elevated levels of asymmetric dimethylarginine (ADMA) may indicate abnormal NO regulation and potentially contribute to endothelial dysfunction in SSc [34]. Additionally, it has been postulated that reduced pulmonary expression of eNOS could be a factor in PAH. Pathological changes in pulmonary tissue may affect the diffusion capacity of NO from the alveolar to vascular space, which could contribute to the reduced FeNO levels observed in PH patients. Furthermore, immune activation has been linked to increased expression of iNOS and elevated levels of circulating NO metabolites in SSc [35, 36]. This suggests that inflammatory mechanisms may play a significant role and potentially mask the reduced NO production by the constitutive endothelial and neuronal NOS isoforms [37].

MCTD-PH may be associated with interstitial lung disease (ILD), and recent literature has increasingly emphasized the significant role of ILD as a contributor to morbidity and mortality among patients with MCTD-PH [38]. To mitigate the potential influence of ILD on FeNO and lung function outcomes, we excluded data from four patients with MCTD who also presented with ILD, the conclusions have not changed (the data were not listed in the table), it suggests that the presence of ILD did not significantly affect the FeNO in severe MCTD-PH patients in the manuscript. This finding indicates that, even in the presence of ILD, FeNO levels remain a reliable and valuable tool for assessing severe MCTD-PH in the manuscript.

This study presents several innovative aspects: (1) We conducted a comprehensive investigation into the distinctions in FeNO levels and lung function between IPAH and MCTD-PH; (2) This study marks the first attempt to establish the correlation between FeNO and lung function parameters such as MEF50%, MEF25%, and MMEF75/25% in severe IPAH. Additionally, we identified an optimal FeNO cutoff value of 23ppb for diagnosing IPAH with severe PH; (3) Furthermore, this research is pioneering in its exploration of the connection between FeNO levels and parameters like R20%, HR, RER, and O2/HR in severe MCTD-PH. However, it’s important to acknowledge potential limitations in our study: (1) We did not collect data from healthy individuals for comparison. Nevertheless, our data still provide valuable insights into the significance of FeNO in severe IPAH and MCTD-PH; (2) Other factors that could influence FeNO measurements were not accounted for, including the consumption of nitrate-rich foods, caffeine and alcohol intake, current smoking status, taking anti-inflammatory drugs, dietary factors, hypereosinophilia and viral infections of the upper and lower respiratory tract. These factors should be considered as potential confounders in future studies.

## Conclusions

This study has uncovered several key findings: In severe IPAH: (1) FeNO exhibited a significant negative correlation with small airway function; (2) Higher FeNO levels were associated with increased peripheral airway obstruction; (3) FeNO levels could effectively diagnose IPAH patients with severe pulmonary hypertension (PH), with an optimal cutoff value of 23ppb. In severe MCTD-PH: (1) FeNO displayed a significant negative correlation with central large airway function and cardiopulmonary function. (2) Lower FeNO levels were linked to higher levels of large airway obstruction, HR, RER, and lower O2/HR. (3) FeNO levels emerged as a crucial marker for identifying severe MCTD-PH. These findings provide valuable insights into the role of FeNO as an indicator of small and large airway function and its potential as a diagnostic tool for severe PH in both IPAH and MCTD-PH.

## Data Availability

All the related data are presented in the manuscript.

## Abbreviations

IPAH: idiopathic pulmonary arterial hypertension
PAH: pulmonary arterial hypertension
MCTD-PH: mixed connective tissue disease complicating pulmonary hypertension
FeNO: Fractional exhaled nitric oxide
pro-BNP: Brain natriuretic peptide
RHC: the right-heart catheterization
CPET: cardiopulmonary exercise test
IOS: impulse Oscillometry
R5: R at 5 Hz
R20: R at 20 Hz
FEV1: Forced Expiratory Velocity at one second
PFT: Pulmonary Function Tests
FVC: forced vital capacity
BMI: body mass index
MEF: maximal expiratory flow
MMEF: maximum mid expiratory flow
IQR: the median
AUC: Area under the ROC curve
WHO: World Health Organization
HR: Heart rate
RER: Respiratory exchange ratio
PETCO2: End-tidal partial pressure for carbon dioxide
PETO2: End-tidal partial pressure for oxygen
NOS: nitric oxide synthase
AT: Anaerobic threshold
ADMA: asymmetric dimethylarginine
SSc: systemic sclerosis
PAH-Scl: pulmonary arterial hypertension related to scleroderma
